# Supplementary material for: HIF1A signaling selectively supports proliferation of breast cancer in the brain
Source: Nat Commun. 2020 Dec 9;11:6311. doi: 10.1038/s41467-020-20144-w (PMC7725834; doi:10.1038/s41467-020-20144-w)
Supplement: Supplementary file 1 — Supplementary Information [file 41467_2020_20144_MOESM1_ESM.pdf]

Supplementary Materials

HIF1A signaling selectively supports proliferation of breast cancer in the brain.

Richard Y. Ebright¥, Marcus A. Zachariah¥, Douglas S. Micalizzi, Ben S. Wittner, Kira L. Niederhoffer, Linda T. Nieman, Brian Chirn, Devon F. Wiley, Benjamin Wesley, Brian Shaw, Edwin Nieblas-Bedolla, Lian Atlas, Annamaria Szabolcs, Anthony J. Iafrate, Mehmet Toner, David T. Ting, Priscilla K. Brastianos, Daniel A. Haber*, Shyamala Maheswaran*

¥ These authors contributed equally.

* Corresponding author. Email: dhaber@mgh.harvard.edu (D.A.H.);

maheswaran@helix.mgh.harvard.edu (S.M.)

This PDF file includes:

Supplementary Figures 1 to 16
Supplementary Data 1 to 7
Supplementary References

Supplementary Figures

Supplementary Figure 1

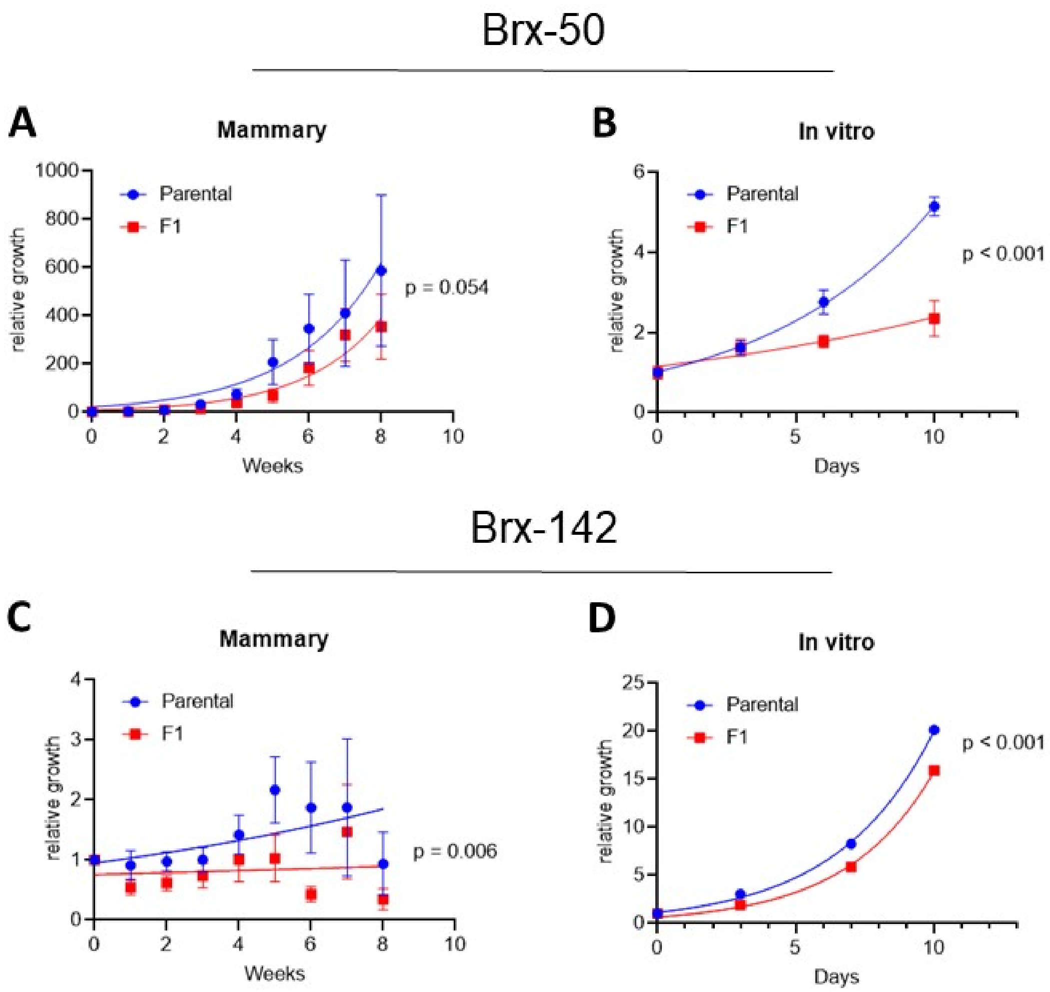

F1 cell lines demonstrate unchanged or decreased mammary and *in vitro* growth: Mammary and *in vitro* growth for Brx-50 (A-B) and Brx-142 (C-D) F1 cell lines (Brx-50: mammary: n = 8; *in vitro*: n = 5 | Brx-142: mammary: n = 10; *in vitro*: n = 5). P values calculated by the extra sum-of-squares F test. Data represent mean values +/- SEM. Source data are provided as a Source Data file.

Supplementary Figure 2

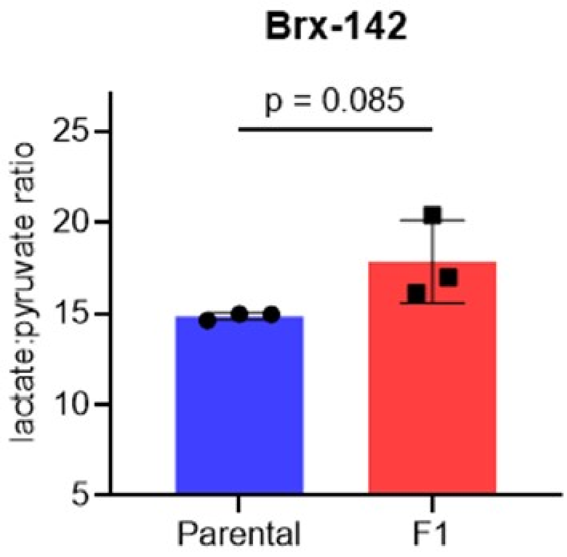

F1 cell lines demonstrate increased glycolysis: Lactate-to-pyruvate ratio of Brx-142 parental and F1 cells, as determined by metabolomic studies for relative levels of polar metabolites (n = 3). P value calculated by two-tailed unpaired t test. Data represent mean values +/- SD. Source data are provided as a Source Data file.

Supplementary Figure 3

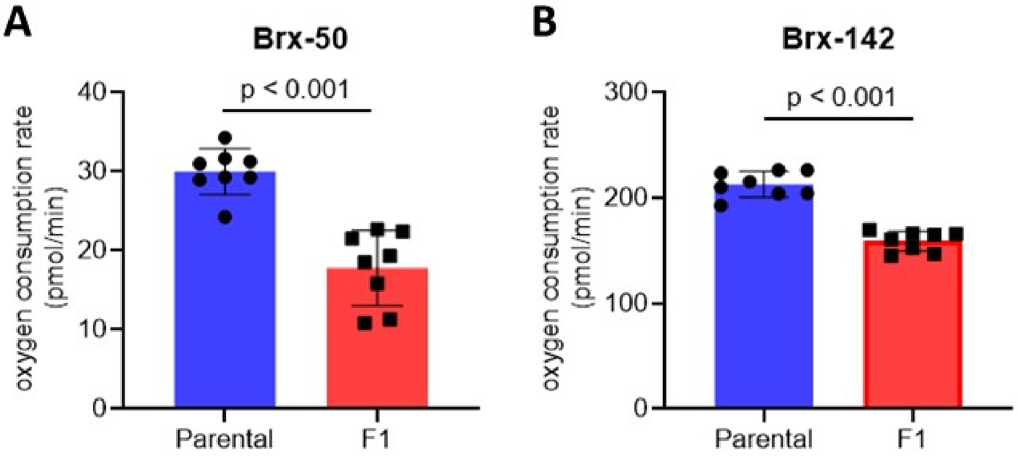

F1 cell lines demonstrate decreased oxidative phosphorylation: Oxygen consumption rate of Brx-50 (A) or Brx-142 (B) parental and F1 cells, as determined by live-cell Seahorse assays (n = 8). P values (Brx-50: $2.28x10^{-5}$; Brx-142: $1.11x10^{-7}$) calculated by two-tailed unpaired t test. Data represent mean values +/- SD. Source data are provided as a Source Data file.

Supplementary Figure 4

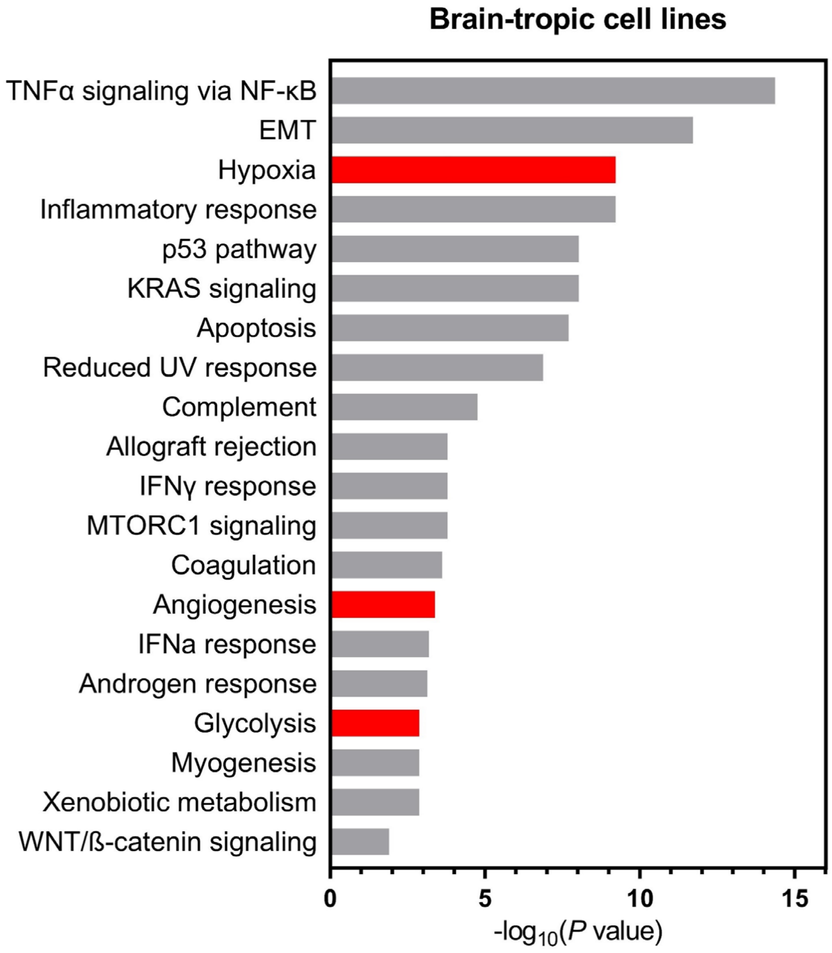

Hypoxic signaling is increased in brain-tropic isogenic ATCC cell lines: GSEA for Hallmarks of Cancer genesets for genes upregulated in brain-tropic MDA-MBA-231 and CN34 cells compared with parental cells (*1*). Hypoxia and downstream pathways of hypoxia (Angiogenesis and Glycolysis) are highlighted.

Supplementary Figure 5

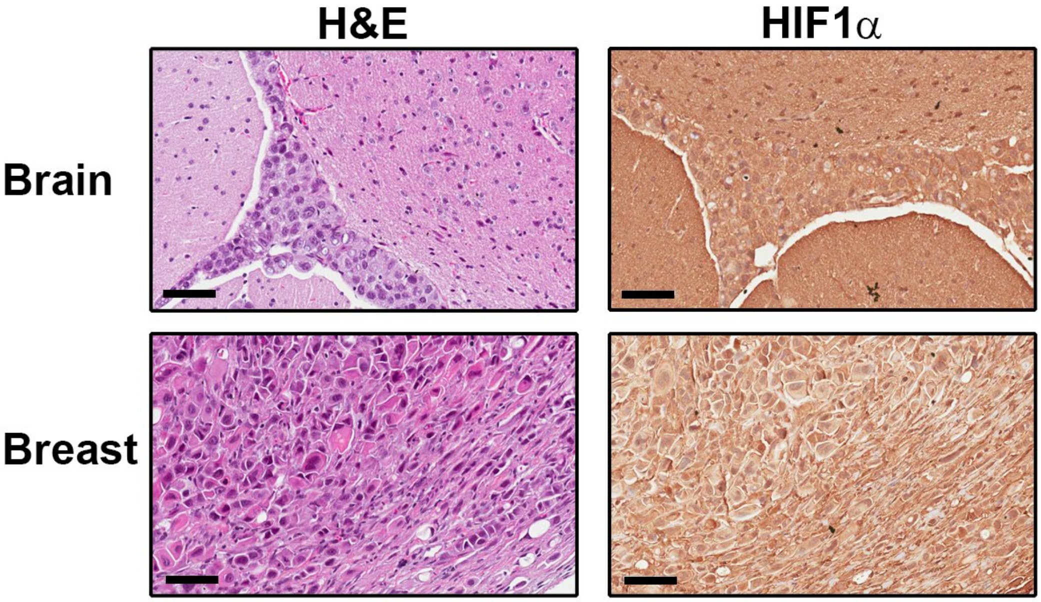

Levels of HIF1A are increased in mouse brain metastases compared with primary breast tumors: Representative sections of Brx-142 brain or breast tumor histology after staining with hematoxylin and eosin; or with anti-HIF1α antibody (brown) and counter-stained with hematoxylin. Scale bars: 70 µm. Images are representative of 4 tumor samples.

Supplementary Figure 6

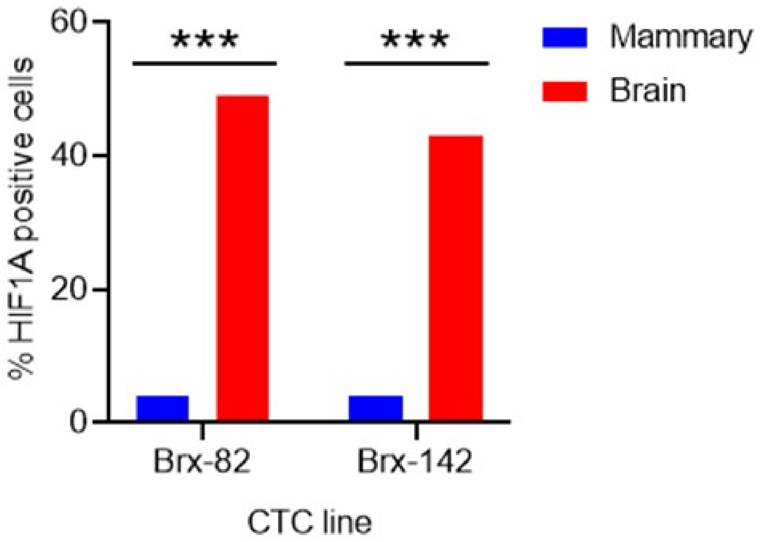

Mouse brain tumors have increased percentages of HIF1A+ cells compared with mammary tumors: Percentage of HIF1A+ cells in Brx-82 and Brx-142 brain and mammary tumors, as determined by automated immunohistochemical staining scoring (n = 1000). P values (Brx-82: $1.05x10^{-115}$; Brx-142: $2.16x10^{-93}$) calculated by two-tailed two population proportion z test. ***: p<0.001. Source data are provided as a Source Data file.

Supplementary Figure 7

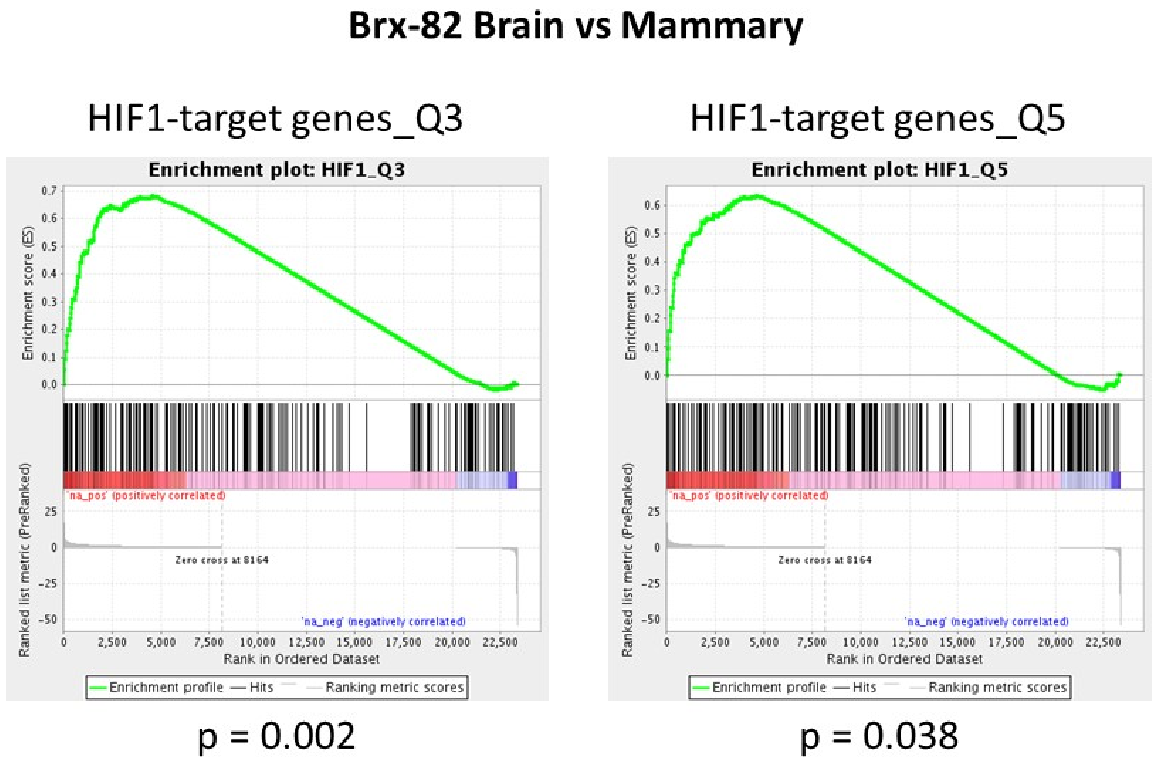

Mouse brain tumors have enrichment for HIF1A target genes compared with mammary tumors: Enrichment plots of the Transcription Factor Targets HIF1_Q3 and HIF1_Q5 genesets for genes enriched in Brx-82 brain tumors versus mammary tumors. Positive enrichment scores in enrichment plots indicate more enrichment of the geneset in brain tumors.

Supplementary Figure 8

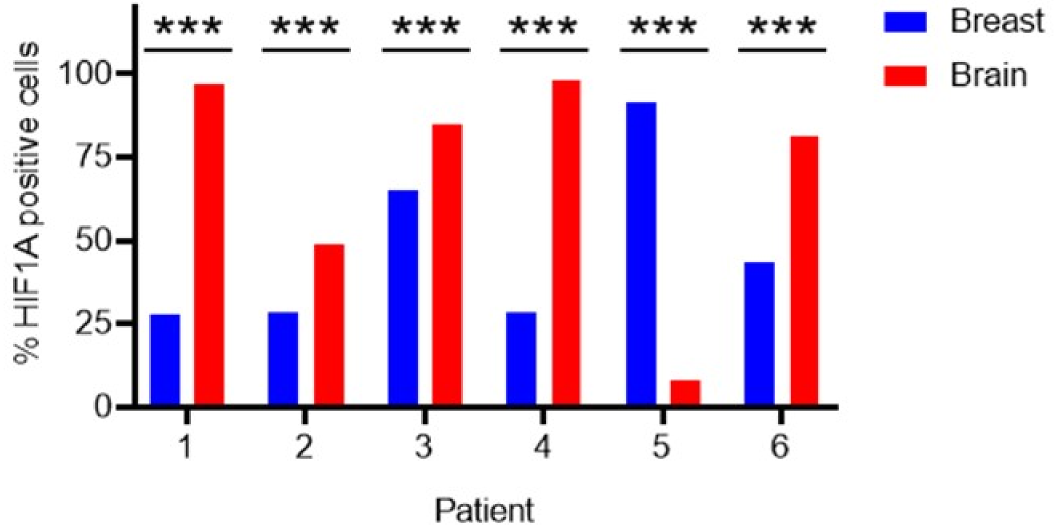

Patient brain metastases have increased percentages of HIF1A+ cells compared with primary breast tumors: Percentage of HIF1A+ cells in matched patient brain metastases and primary breast tumors, as determined by automated immunohistochemical staining scoring (Patient #3 breast: n = 262; all other samples: n = 1000). P values (Patient 1: $<1x10^{-300}$; Patient 2: $4.52x10^{-122}$; Patient 3: $7.02x10^{-24}$; Patient 4: $3.59x10^{-227}$; Patient 5: $<1x10^{-300}$; Patient 6: $6.83x10^{-69}$) calculated by two-tailed two population proportion z test. ***: p<0.001. Source data are provided as a Source Data file.

Supplementary Figure 9

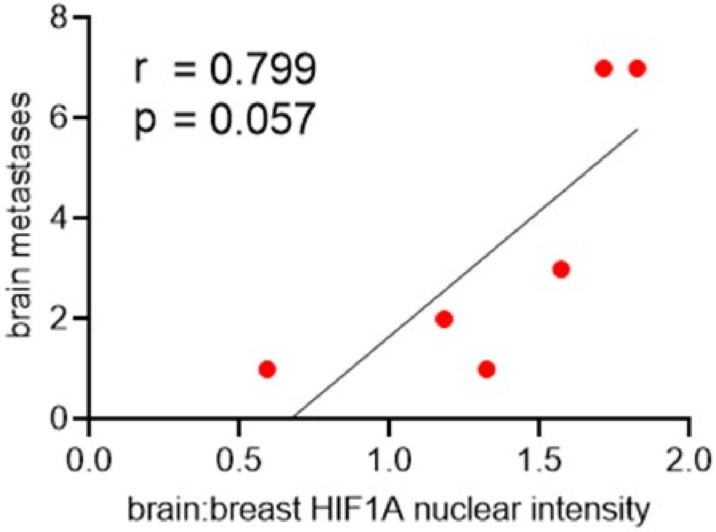

Increased patient brain metastasis versus breast primary HIF1A staining predicts increased number of brain metastases: Ratio of matched patient brain-to-breast HIF1A nuclear intensity positively correlates to number of detected brain metastases. Line of best fit displayed. Correlation calculated as Pearson's correlation coefficient. P value calculated by two-tailed unpaired t test.

Supplementary Figure 10

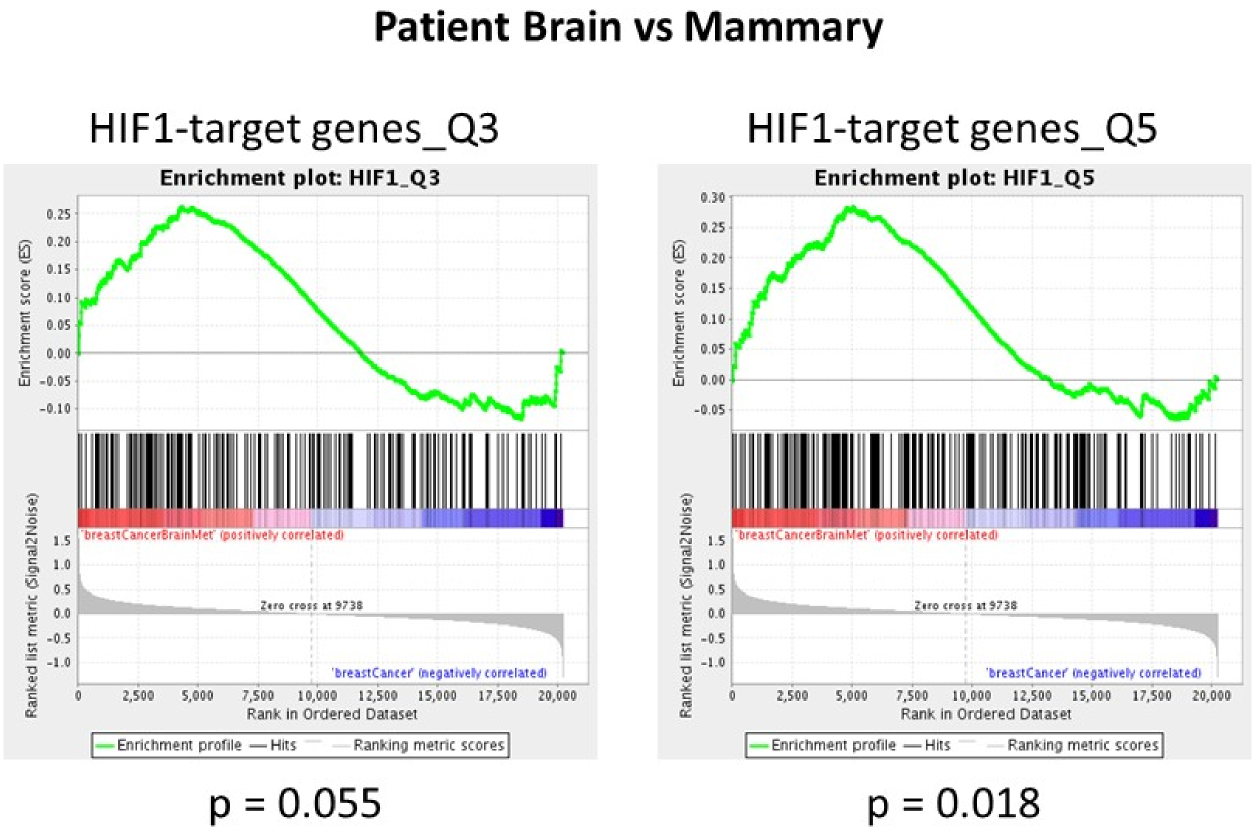

Patient brain metastases have enrichment for HIF1A target genes compared with primary breast tumors: Enrichment plots of the Transcription Factor Targets HIF1_Q3 and HIF1_Q5 genesets for genes enriched in patient brain metastases versus unmatched primary breast tumors (*2*). Positive enrichment scores in enrichment plots indicate more enrichment of the geneset in brain metastases.

Supplementary Figure 11

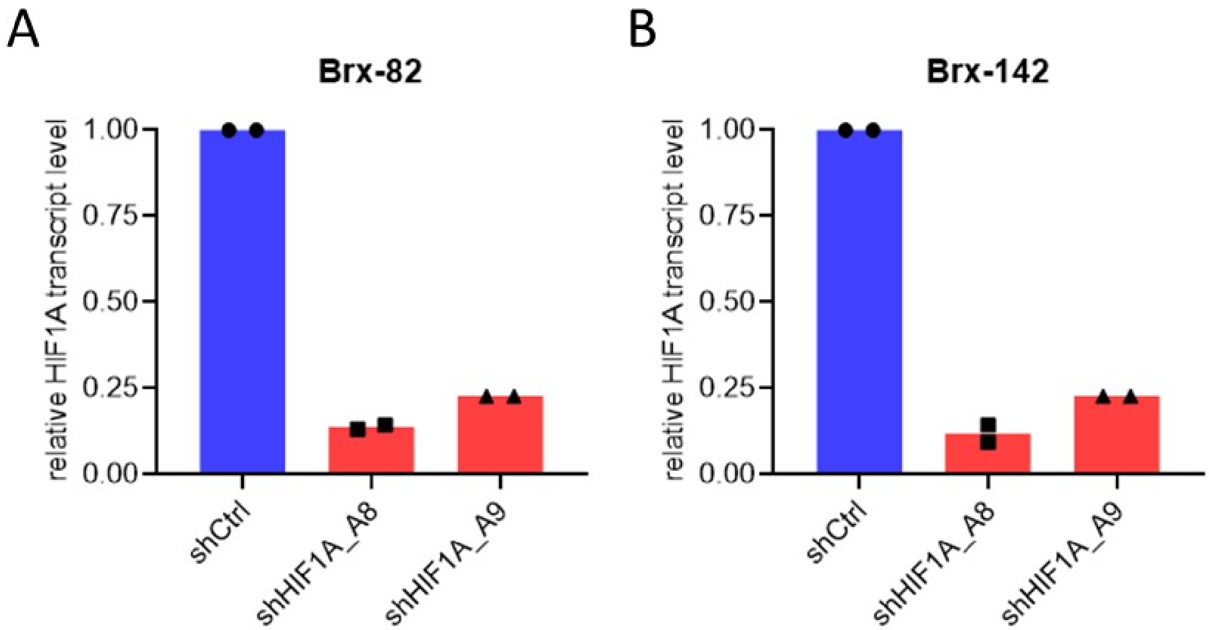

Knockdown of HIF1A using shRNAs: qPCR for *HIF1A* in Brx-82 (A) and Brx-142 (B) cells following small hairpin knockdown of shHIF1A (using either hairpin A8 or A9) (n = 2). Source data are provided as a Source Data file.

Supplementary Figure 12

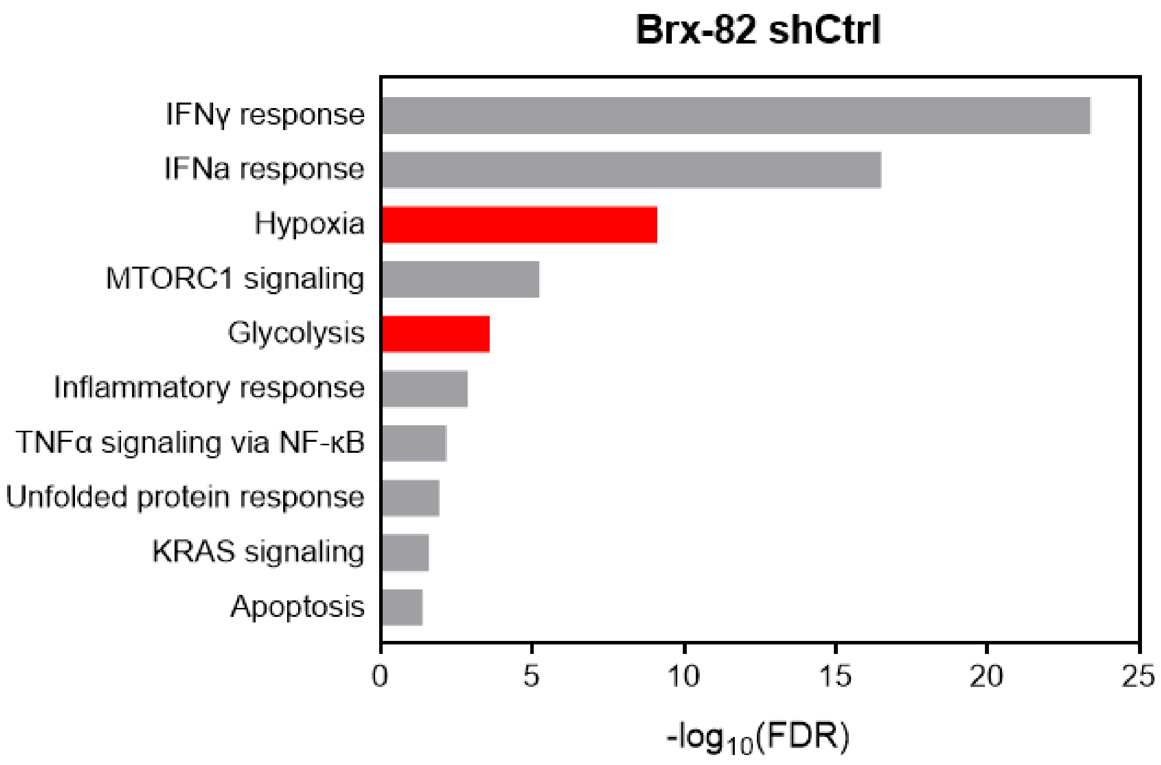

HIF1A knockdown decreases hypoxia and glycolytic signaling: GSEA of transcripts differentially expressed in Brx-82 shCtrl compared with shHIF1A_A8 cells (fold change > 2; FDR < 0.25). The most enriched Hallmarks of Cancer gene sets from the Broad Molecular Signatures Database and associated FDR values are shown. Hypoxia and Glycolysis genesets are highlighted.

Supplementary Figure 13

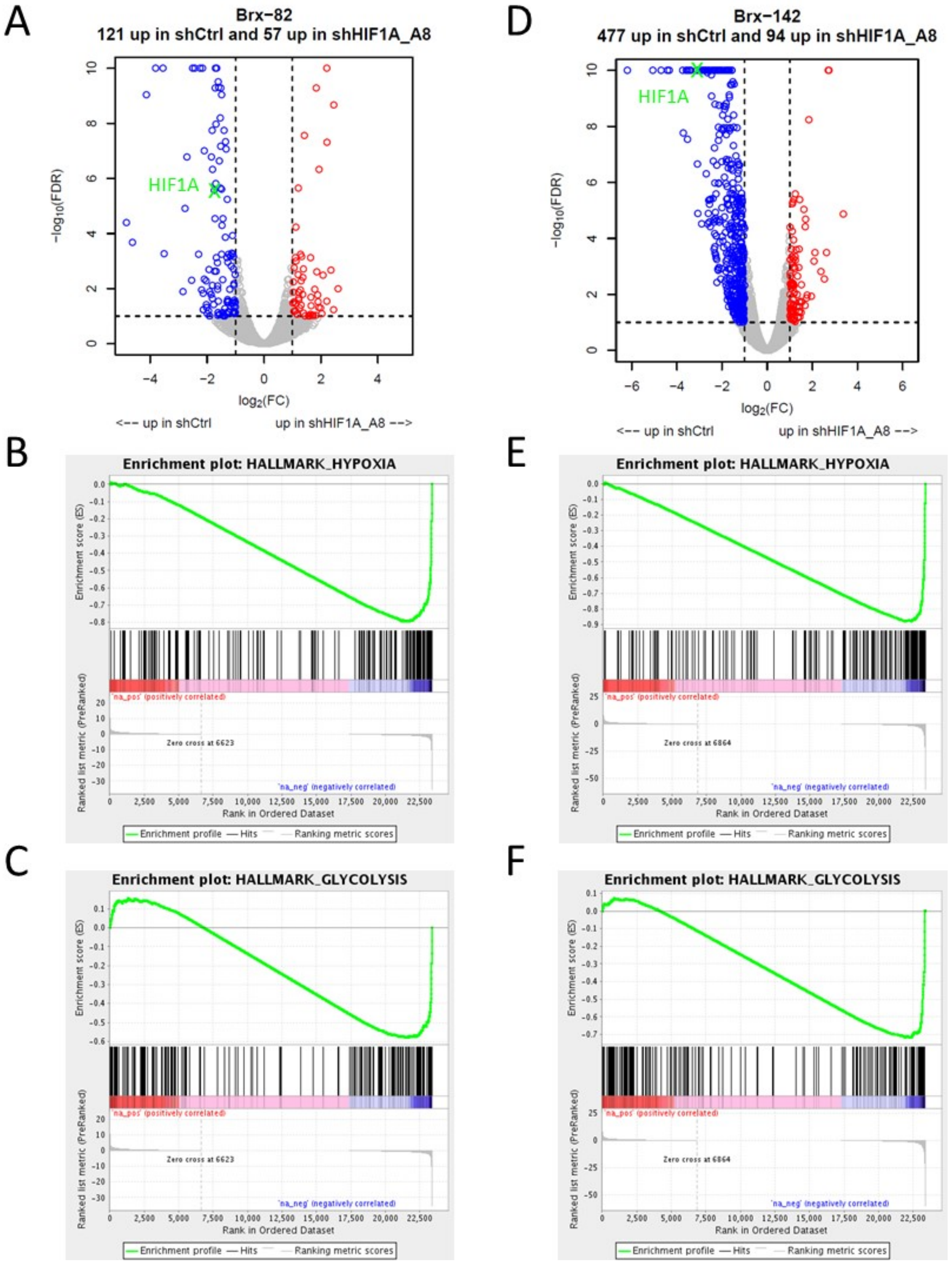

HIF1A knockdown decreases hypoxia and glycolytic signaling: Knockdown of HIF1A in Brx-82 (A-C) and Brx-142 (D-F) cells leads to significant changes in the transcriptome and decreased hypoxic and glycolytic signaling. Blue indicates higher expression in shCtrl cells and red indicates higher expression in shHIF1A_8 cells (FC > 2; FDR < 0.25). Genes with -$\log_{10}$(FDR) > 10 are displayed as -$\log_{10}$(FDR) = 10. Negative enrichment scores in enrichment plots indicate more enrichment of the geneset in shCtrl cells.

Supplementary Figure 14

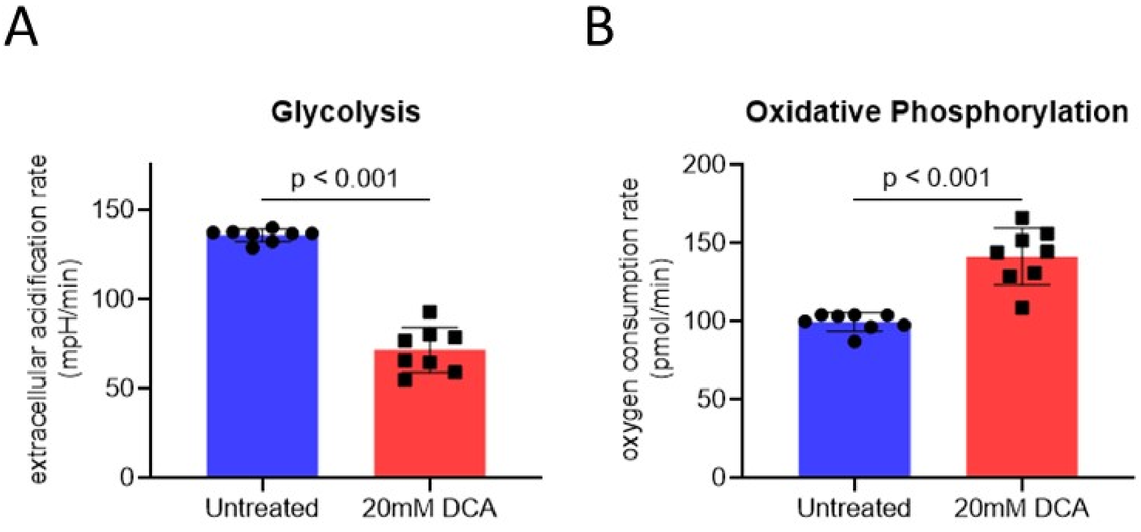

Dichloroacetic acid treatment inhibits glycolysis: Extracellular acidification rate (A) and oxygen consumption rate (B) of Brx-82 cells treated with 20mM dichloroacetic acid (DCA) or with vehicle, as determined by live-cell Seahorse assays (n = 8). P values (Glycolysis: $1.54x10^{-9}$; Oxidative phosphorylation: $2.31x10^{-5}$) calculated by two-tailed unpaired t test. Data represent mean values +/- SD. Source data are provided as a Source Data file.

Supplementary Figure 15

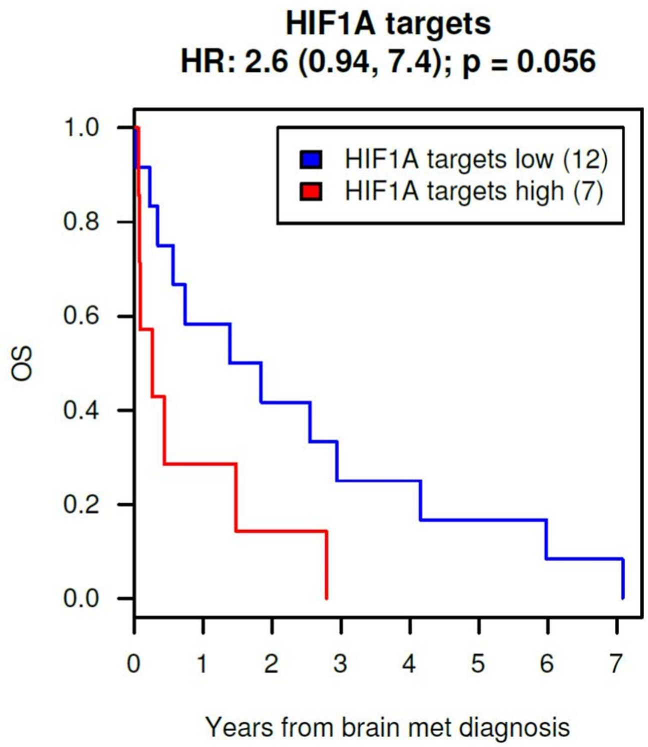

HIF1A signaling in CTCs predicts poor outcome in brain metastasis patients: Kaplan-Meier analysis of the OS following brain metastasis diagnosis for patients with high average HIF1A target gene expression in CTCs versus those with low average HIF1A target gene expression (Transcription Factor Targets HIF1_Q3 geneset). The HIF1A targets-high and HIF1A targets-low subgroups were determined on the basis of average HIF1A target gene expression across all CTCs isolated for each patient. P value calculated by log rank test.

Supplementary Figure 16

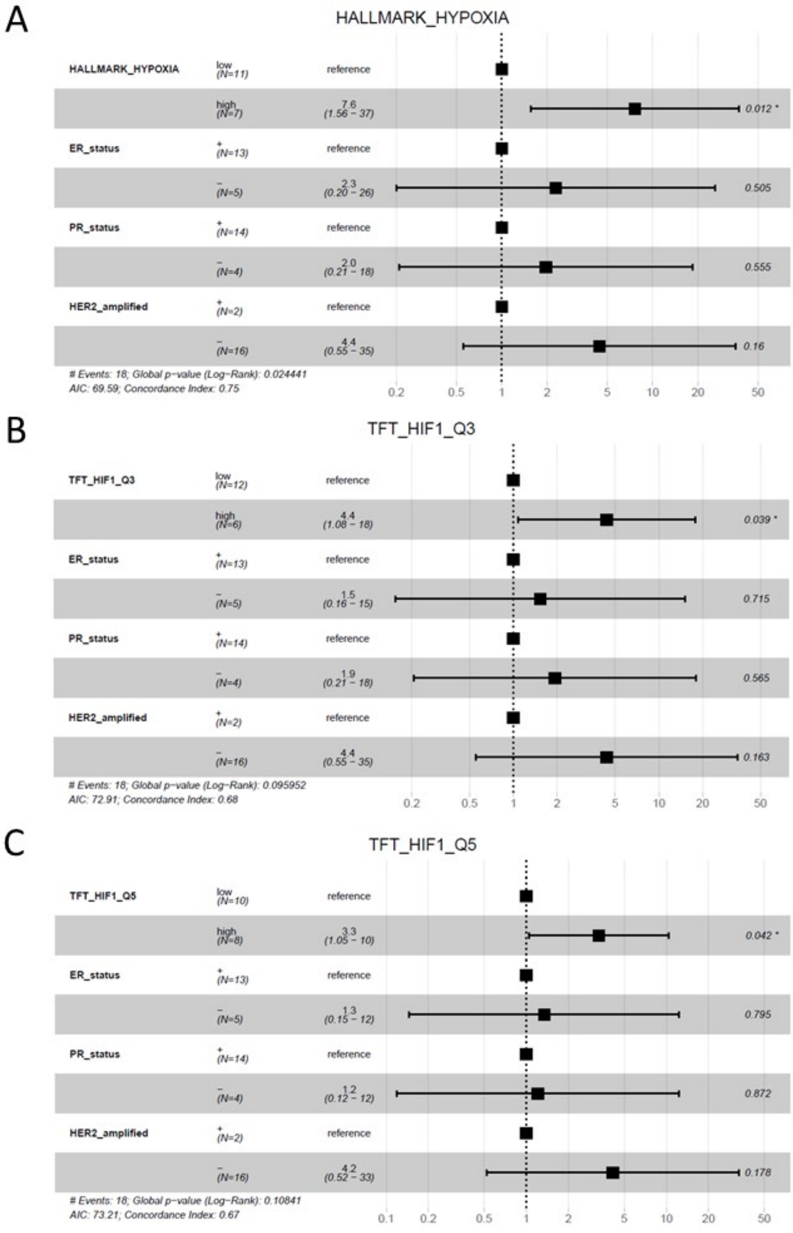

Correlation of CTC hypoxic signaling to overall survival is independent of breast cancer subtype: Multivariate Cox proportional hazards modeling of OS after brain metastasis diagnosis by ER, PR, and HER2 status together with Hallmark Hypoxia (A), Transcription Factor Targets HIF1_Q3 (B) and Transcription Factor Targets HIF1_Q5 (C) geneset expression levels. Brx-206 was excluded due to unknown HER2 status.

Supplementary Data

Supplementary Data 1

CTC patient	Histology	ER status	PR status	HER2 amplified	Intracranial metastasis
**Brx-7**	Lobular	+	+	-	-
**Brx-29**	Lobular	+	+	-	+
**Brx-42**	Ductal/Lobular	+	+	-	+
**Brx-50**	Ductal	+	+	-	-
**Brx-68**	Ductal	+	-	-	-
**Brx-82**	Lobular	+	+	-	+
**Brx-142**	Lobular	+	+	-	-

Patient clinical characteristics for seven parental CTC lines. No patients overlap with patients shown in Supplementary Data 4. Patients Brx-42 and Brx-82 overlap with patients shown in Supplementary Data 6.

Supplementary Data 2

CTC line	Gene	Type	Effect	Result	Allele_freq
Brx-50 P	CDH1	deletion	frameshift_variant	p.Asp288ArgfsTer2	0.862
Brx-50 P	ESR1	SNV	missense	p.Leu536Pro	0.423
Brx-50 P	JAK3	SNV	missense	p.Arg403Cys	0.385
Brx-50 F1	CDH1	deletion	frameshift_variant	p.Asp288ArgfsTer2	0.912
Brx-50 F1	ESR1	SNV	missense	p.Leu536Pro	0.493
Brx-50 F1	JAK3	SNV	missense	p.Arg403Cys	0.609
Brx-82 P	ERBB2	SNV	missense	p.Ser310Tyr	1
Brx-82 P	TP53	SNV	missense	p.Arg248Gln	0.725
Brx-82 P	KEAP1	SNV	missense	p.Ala321Thr	0.688
Brx-82 P	ERBB2	SNV	missense	p.Glu238Lys	0.719
Brx-82 P	PIK3R1	insertion	frameshift_variant	p.Leu347ThrfsTer17	0.188
Brx-82 F1	ERBB2	SNV	missense	p.Ser310Tyr	1
Brx-82 F1	TP53	SNV	missense	p.Arg248Gln	0.712
Brx-82 F1	KEAP1	SNV	missense	p.Ala321Thr	0.728
Brx-82 F1	ERBB2	SNV	missense	p.Glu238Lys	0.772
Brx-82 F1	ALK *	SNV	missense	p.Gln39Pro	0.518
Brx-82 F1	PIK3R1	insertion	frameshift_variant	p.Leu347ThrfsTer17	0.22
Brx-82 F2	ERBB2	SNV	missense	p.Ser310Tyr	1
Brx-82 F2	TP53	SNV	missense	p.Arg248Gln	0.649
Brx-82 F2	KEAP1	SNV	missense	p.Ala321Thr	0.668
Brx-82 F2	ERBB2	SNV	missense	p.Glu238Lys	0.595
Brx-82 F2	PIK3R1	insertion	frameshift_variant	p.Leu347ThrfsTer17	0.211
Brx-142 P	PIK3CA	SNV	missense	p.His1047Arg	0.347
Brx-142 P	TP53	SNV	stop_gained	p.Glu349Ter	1
Brx-142 P	TERT	SNV	upstream_gene_variant	null	0.389
Brx-142 P	MAP2K1	SNV	stop_gained	p.Glu69Ter	0.448
Brx-142 P	CDH1	SNV	splice_donor_variant	null	0.991
Brx-142 P	ARID1A	SNV	stop_gained	p.Gln1066Ter	0.631
Brx-142 P	ATM	SNV	missense	p.Phe627Cys	0.496
Brx-142 F1	PIK3CA	SNV	missense	p.His1047Arg	0.293
Brx-142 F1	TP53	SNV	stop_gained	p.Glu349Ter	0.995
Brx-142 F1	TERT	SNV	upstream_gene_variant	null	0.399
Brx-142 F1	MAP2K1	SNV	stop_gained	p.Glu69Ter	0.442
Brx-142 F1	CDH1	SNV	splice_donor_variant	null	1
Brx-142 F1	ARID1A	SNV	stop_gained	p.Gln1066Ter	0.57
Brx-142 F1	ATM	SNV	missense	p.Phe627Cys	0.497

DNA-sequencing of Brx-50, Brx-82, and Brx-142 parental (P), F1 and F2 cell lines.

*** indicates a mutation in Brx-82 F1 cells not observed in Brx-82 parental or F2 cells.**

Supplementary Data 3

Enriched in brain tumors	
**TFT geneset**	**NOM p-val**
E2F1DP2_01	< 0.001
E2F_02	< 0.001
TGASTMAGC_NFE2_01	< 0.001
E2F1DP1_01	< 0.001
E2F4DP2_01	< 0.001
E2F1_Q6	< 0.001
E2F_Q6	< 0.001
E2F_Q4	< 0.001
E2F4DP1_01	< 0.001
E2F1_Q4_01	< 0.001
E2F1_Q3	< 0.001
E2F_Q3_01	< 0.001
E2F1_Q6_01	< 0.001
E2F_Q4_01	< 0.001
E2F1DP1RB_01	< 0.001
E2F1_Q3_01	< 0.001
**HIF1_Q3**	**0.002**
E2F_03	0.002
E2F_Q3	0.003
E2F_Q6_01	0.003
E2F1_Q4	0.005
RAAGNYNNCTTY_UNKNOWN	0.007
ALPHACP1_01	0.007
SCGGAAGY_ELK1_02	0.009
NFY_Q6	0.010
ELK1_02	0.010
ACCTGTTG_UNKNOWN	0.011
GCTNWTTGK_UNKNOWN	0.023
CDPCR3_01	0.033
**HIF1_Q5**	**0.038**
CTTTGT_LEF1_Q2	0.043
ARNT_01	0.048
NRF1_Q6	0.048
USF_01	0.049
AACYNNNNTTCCS_UNKNOWN	0.050
NFY_01	0.058
AHR_Q5	0.060
PAX_Q6	0.060
SREBP1_01	0.062
AP2_Q6	0.069
AACTTT_UNKNOWN	0.071
RTTTNNNYTGGM_UNKNOWN	0.083
NMYC_01	0.087
CGTSACG_PAX3_B	0.089
GCGNNANTTCC_UNKNOWN	0.092
WHN_B	0.092
EFC_Q6	0.096
TTANTCA_UNKNOWN	0.097

GSEA of RNA-seq from Brx-82 brain and mammary tumors for pathways enriched within the Molecular Signatures Database Transcription Factor Targets genesets, showing genesets enriched in brain tumors. HIF1A genesets highlighted.

Supplementary Data 4

BCBM patient	Years between primary and brain metastasis diagnoses	ER status	PR status	HER2 amplified	Number intracranial metastases
1	2.6	+	+	-	7
2	10.8	+	+	+	2
3	4.2	+	+	-	3
4	2.6	+	-	+	7
5	2.1	-	-	+	1
6	15.2	-	+	-	1

Patient clinical characteristics for six matched primary breast and brain metastasis samples. No patients overlap with patients shown in Supplementary Data 1 or Supplementary Data 6. BCBM: breast cancer brain metastasis.

Supplementary Data 5

Enriched in brain metastases	
**TFT geneset**	**NOM p-val**
AP2_Q3	0.006
E2F_Q3	0.007
E2F_Q4_01	0.015
**HIF1_Q5**	**0.018**
GGARNTKYCCA_UNKNOWN	0.021
GNCF_01	0.021
SREBP1_02	0.023
E2F1_Q6_01	0.025
AACWWCAANK_UNKNOWN	0.025
SF1_Q6	0.027
YNTTTNNNANGCARM_UNKNOWN	0.027
E2F_Q3_01	0.028
ETF_Q6	0.031
GGATTA_PITX2_Q2	0.042
E2F1_Q4_01	0.042
E2F1_Q6	0.054
**HIF1_Q3**	**0.055**
NFY_Q6_01	0.056
CDPCR3_01	0.057
E2F1_Q3	0.072
GGAMTNNNNNTCCY_UNKNOWN	0.074
GATA1_01	0.082
E2F4DP1_01	0.087
ER_Q6_02	0.089
E2F1DP2_01	0.092
E2F_Q6_01	0.096

GSEA of RNA-seq from patient brain metastasis and unmatched primary breast tumors (*2*) for pathways enriched within the Molecular Signatures Database Transcription Factor Targets genesets, showing genesets enriched in brain metastases. HIF1A genesets highlighted.

Supplementary Data 6

patient	CTC ID	ER status	PR status	HER2 amplified	Patient draw number	OS after brain met dx
BR29	BR29_1_012913	+	+	-	1	9
BR29	BR29_2_012913	+	+	-	1	9
BR29	BR29_3_BYL_012913	+	+	-	1	9
Brx-10	Brx10_1_SC1_041113	+	+	-	1	1071
Brx-10	Brx10_1_SC2_041113	+	+	-	1	1071
Brx-10	Brx10_1_SC3_041113	+	+	-	1	1071
Brx-110	BRx-110-3--06-29-15	+	+	-	1	206
Brx-110	BRx-110-2--9-8	+	+	-	2	206
Brx-110	BRx-110-3--9-8	+	+	-	2	206
Brx-110	BRx-110-1--09-17-15	+	+	-	3	206
Brx-110	BRx-110-2--09-17-15	+	+	-	3	206
Brx-110	BRx-110-1--10-13-15	+	+	-	4	206
Brx-110	BRx-110-2--10-13-15	+	+	-	4	206
Brx-111	BRx-111-1--10-08-15	-	-	-	1	672
Brx-111	BRx-111-2--10-08-15	-	-	-	1	672
Brx-111	BRx-111-3--10-08-15	-	-	-	1	672
Brx-111	BRx-111-1--10-19-15	-	-	-	2	672
Brx-122	Brx122_1_SC1_121213	+	+	-	1	157
Brx-122	Brx122_1_SC2_121213	+	+	-	1	157
Brx-122	Brx122_1_SC3_121213	+	+	-	1	157
Brx-129	BRx-129-2--9-29	-	-	-	1	267
Brx-146	Brx146_2_relapse_SC1_082114	-	+	-	1	930
Brx-146	Brx146_2_relapse_SC2_082114	-	+	-	1	930
Brx-146	Brx146_2_relapse_SC3_082114	-	+	-	1	930
Brx-156	BRx-156-2--12-01-15	+	+	-	1	124
Brx-172	BRx-172_2_111114	-	-	-	1	506
Brx-172	BRx-172-5--11-11-14	-	-	-	1	506
Brx-172	BRx-172-1--02-01-15	-	-	-	2	506
Brx-172	BRx-172-1--4-16-15	-	-	-	3	506
Brx-172	BRx-172-2--07-07-15	-	-	-	4	506
Brx-172	BRx-172-3--07-07-15	-	-	-	4	506
Brx-172	BRx-172-1-08-04-15	-	-	-	5	506
Brx-172	BRx-172-1--08-18-15	-	-	-	6	506
Brx-172	BRx-172-1--08-25-15	-	-	-	7	506
Brx-172	BRx-172-2--08-25-15	-	-	-	7	506
Brx-172	BRx-172-1--09-09-15	-	-	-	8	506
Brx-172	BRx-172-1--9-30	-	-	-	9	506
Brx-18	BRx-18du42	+	+	+	1	2587

Brx-206	BRx-206-1--10-23-15	+	+	unknown	1	29
Brx-206	BRx-206-2--10-23-15	+	+	unknown	1	29
Brx-206	BRx-206-1--01-15-16	+	+	unknown	2	29
Brx-206	BRx-206-2--01-15-16	+	+	unknown	2	29
Brx-206	BRx-206-3--01-15-16	+	+	unknown	2	29
Brx-206	BRx-206-4--01-15-16	+	+	unknown	2	29
Brx-221	BRx-221-1--10-13-15	+	+	-	1	2184
Brx-221	BRx-221-2--10-13-15	+	+	-	1	2184
Brx-245	BRx-245-2--09-04-15	-	-	-	1	80
Brx-42	BRx-42-1--9-14	+	+	-	1	28
Brx-42	BRx-42-2--9-14	+	+	-	1	28
Brx-42	BRx-42-1--9-22	+	+	-	2	28
Brx-42	BRx-42-1--9-29	+	+	-	3	28
Brx-42	BRx-42-2--9-29	+	+	-	3	28
Brx-42	BRx-42-3--9-29	+	+	-	3	28
Brx-42	BRx-42-1--10-20	+	+	-	4	28
Brx-42	BRx-42_2_110314	+	+	-	5	28
Brx-74	Brx74_1_SC2_032813	+	+	-	1	21
Brx-82	Brx82_1_SC1_051613	+	+	-	1	94
Brx-82	BRx-82-2--9-15	+	+	-	2	94
Brx-82	BRx-82-1--9-29	+	+	-	3	94
Brx-82	BRx-82-2--9-29	+	+	-	3	94
Brx-82	BRx-82-1--11-13-14	+	+	-	4	94
Brx-82	BRx-82-2--11-13-14	+	+	-	4	94
Brx-82	BRx-82_1_012015	+	+	-	5	94
Brx-82	BRx-82-1--1-12-15	+	+	-	5	94
Brx-82	BRx-82-3--01-20-15	+	+	-	5	94
Brx-82	BRx-82-4--01-20-15	+	+	-	5	94
Brx-82	BRx-82_1_030915	+	+	-	6	94
Brx-82	BRx-82_2_030915	+	+	-	6	94
Brx-82	BRx-82_3_030915	+	+	-	6	94
Brx-82	BRx-82_5_030915	+	+	-	6	94
Brx-82	BRx-82_6_030915	+	+	-	6	94
Brx-82	BRx-82_7_030915	+	+	-	6	94
Brx-82	BRx-82-8--3-9-15	+	+	-	6	94
Brx-86	Brx86_1_CL1_052313	+	+	+	1	539
Brx-86	Brx86_1_SC2_052313	+	+	+	1	539
Brx-86	Brx86_1_SC3_052313	+	+	+	1	539
Brx-86	Brx86_2_BYL_SC1_052413	+	+	+	2	539
Brx-86	Brx86_2_BYL_SC2_052413	+	+	+	2	539
Brx-86	Brx86_2_BYL_SC3_052413	+	+	+	2	539
Brx-87	Brx87_1_SC1_062113	+	+	-	1	1016
Brx-87	Brx87_1_SC2_062113	+	+	-	1	1016

Brx-95	Brx95_1_SC1_071713	+	+	-	1	1517
Brx-95	Brx95_2_BYL_CL1_071813	+	+	-	2	1517

Patient clinical characteristics for 83 patient-derived CTCs from 19 patients with brain metastases. Patients Brx-42 and Brx-82 overlap with patients shown in Supplementary Data 1. No patients overlap with patients shown in Supplementary Data 4. All patients were deceased at last follow-up.

Supplementary Data 7

Primer	Sequence/Assay ID
shCtrl_hairpin	CAACAAGATGAAGAGCACCAA
shHIF1A_A8_hairpin	GTGATGAAAGAATTACCGAAT
shHIF1A_A9_hairpin	CGGCGAAGTAAAGAATCTGAA
shCtrl_sequencing	CCGGCAACAAGATGAAGAGCACCAACTCGAGTTGGTGCTCTTCATCTTGTTGTTTTT
shHIF1A_A8_sequencing	CCGGGTGATGAAAGAATTACCGAATCTCGAGATTCGGTAATTCTTTCATCACTTTTT
shHIF1A_A9_sequencing	CCGGCGGCGAAGTAAAGAATCTGAACTCGAGTTCAGATTCTTTACTTCGCCGTTTTT
ACTB_TaqMan	Hs01060665_g1
HIF1A_TaqMan	Hs00153153_m1

Primers used for shRNA knockdown and sequencing.

Supplementary References

1. P. D. Bos *et al.*, Genes that mediate breast cancer metastasis to the brain. *Nature* **459**, 1005-1009 (2009).
2. H. J. Schulten *et al.*, Comprehensive molecular biomarker identification in breast cancer brain metastases. *J Transl Med* **15**, 269 (2017).
