# Supplementary material for: HIF1A signaling selectively supports proliferation of breast cancer in the brain
Source: Nat Commun. 2020 Dec 9;11:6311. doi: 10.1038/s41467-020-20144-w (PMC7725834; doi:10.1038/s41467-020-20144-w)
Supplement: Supplementary file 3 — Description of Additional Supplementary Files [file 41467_2020_20144_MOESM3_ESM.docx]

File Name: Supplementary Data 1

Description: **Patient clinical characteristics for seven parental CTC lines. No patients overlap with patients shown in Supplementary Data 4. Patients Brx-42 and Brx-82 overlap with patients shown in Supplementary Data 6.**

File Name: Supplementary Data 2

Description: **DNA-sequencing of Brx-50, Brx-82, and Brx-142 parental (P), F1 and F2 cell lines. * indicates a mutation in Brx-82 F1 cells not observed in Brx-82 parental or F2 cells.**

File Name: Supplementary Data 3

Description: **GSEA of RNA-seq from Brx-82 brain and mammary tumors for pathways enriched within the Molecular Signatures Database Transcription Factor Targets genesets, showing genesets enriched in brain tumors. HIF1A genesets highlighted.**

File Name: Supplementary Data 4

Description: **Patient clinical characteristics for six matched primary breast and brain metastasis samples. No patients overlap with patients shown in Supplementary Data 1 or Supplementary Data 6. BCBM: breast cancer brain metastasis.**

File Name: Supplementary Data 5

Description: **GSEA of RNA-seq from patient brain metastasis and unmatched primary breast tumors (*2*) for pathways enriched within the Molecular Signatures Database Transcription Factor Targets genesets, showing genesets enriched in brain metastases. HIF1A genesets highlighted.**

File Name: Supplementary Data 6

Description: **Patient clinical characteristics for 83 patient-derived CTCs from 19 patients with brain metastases. Patients Brx-42 and Brx-82 overlap with patients shown in Supplementary Data 1. No patients overlap with patients shown in Supplementary Data 4. All patients were deceased at last follow-up.**

File Name: Supplementary Data 7

Description: **Primers used for shRNA knockdown and sequencing.**
