# Supplementary material for: HIF1A signaling selectively supports proliferation of breast cancer in the brain
Source: Nat Commun. 2020 Dec 9;11:6311. doi: 10.1038/s41467-020-20144-w (PMC7725834; doi:10.1038/s41467-020-20144-w)
Supplement: Supplementary file 11 — Reporting Summary [file 41467_2020_20144_MOESM11_ESM.pdf]

nature research

Corresponding author(s): Daniel Haber, Shyamala Maheswaran
Last updated by author(s): Oct 21, 2020

# Reporting Summary

Nature Research wishes to improve the reproducibility of the work that we publish. This form provides structure for consistency and transparency in reporting. For further information on Nature Research policies, see our Editorial Policies and the Editorial Policy Checklist.

## Statistics

For all statistical analyses, confirm that the following items are present in the figure legend, table legend, main text, or Methods section.

| n/a | Confirmed | |
|---|---|---|
| ☐ | ☒ | The exact sample size ($n$) for each experimental group/condition, given as a discrete number and unit of measurement |
| ☐ | ☒ | A statement on whether measurements were taken from distinct samples or whether the same sample was measured repeatedly |
| ☐ | ☒ | The statistical test(s) used AND whether they are one- or two-sided<br>*Only common tests should be described solely by name; describe more complex techniques in the Methods section.* |
| ☐ | ☒ | A description of all covariates tested |
| ☐ | ☒ | A description of any assumptions or corrections, such as tests of normality and adjustment for multiple comparisons |
| ☐ | ☒ | A full description of the statistical parameters including central tendency (e.g. means) or other basic estimates (e.g. regression coefficient) AND variation (e.g. standard deviation) or associated estimates of uncertainty (e.g. confidence intervals) |
| ☐ | ☒ | For null hypothesis testing, the test statistic (e.g. $F$, $t$, $r$) with confidence intervals, effect sizes, degrees of freedom and $P$ value noted<br>*Give P values as exact values whenever suitable.* |
| ☒ | ☐ | For Bayesian analysis, information on the choice of priors and Markov chain Monte Carlo settings |
| ☒ | ☐ | For hierarchical and complex designs, identification of the appropriate level for tests and full reporting of outcomes |
| ☐ | ☒ | Estimates of effect sizes (e.g. Cohen's $d$, Pearson's $r$), indicating how they were calculated |

*Our web collection on statistics for biologists contains articles on many of the points above.*

## Software and code

Policy information about availability of computer code

| | |
|---|---|
| Data collection | No software was used for data collection. |
| Data analysis | The R programming language, versions 3.1.2 and 3.6.1, GSEA software, the Bioconductor edgeR package, and the DESEQ2 package were used for data analysis. HALO Image Analysis software was used for immunohistochemical analysis. PRISM was used for data visualization. |

For manuscripts utilizing custom algorithms or software that are central to the research but not yet described in published literature, software must be made available to editors and reviewers. We strongly encourage code deposition in a community repository (e.g. GitHub). See the Nature Research guidelines for submitting code & software for further information.

## Data

Policy information about availability of data

All manuscripts must include a data availability statement. This statement should provide the following information, where applicable:

- Accession codes, unique identifiers, or web links for publicly available datasets
- A list of figures that have associated raw data
- A description of any restrictions on data availability

Raw data from RNA-seq of CTC cultures and CTC-derived mouse xenograft mammary and brain tumors have been deposited in the Gene Expression Omnibus (GEO) database under accession number GSE156944. These data correspond to Figures 3A-D, 4C, 5B, S7, S10, S12, and S13, as well as Tables 1, S3, and S5. Additionally, data from GSE12237 are used in Figure S4, data from GSE100534 are used in Figure 4F, and data combined from GSE144494 and GSE144495 are used in Figures 6A-C, S15, and S16.

# Field-specific reporting

Please select the one below that is the best fit for your research. If you are not sure, read the appropriate sections before making your selection.

- [x] Life sciences
- [ ] Behavioural & social sciences
- [ ] Ecological, evolutionary & environmental sciences

For a reference copy of the document with all sections, see nature.com/documents/nr-reporting-summary-flat.pdf

# Life sciences study design

All studies must disclose on these points even when the disclosure is negative.

| | |
|---|---|
| Sample size | Sample size was chosen based on a standard power calculation required to have 90% confidence in detecting a >10 fold signal modulation (B = 0.9; standard dev 50% baseline signal; type 1 error rate 0.05). This yielded 4 biological replicates/sample. For brain injection experiments, an additional 2-4 mice were added for brain injection experiments, as stereotactic brain injections in our hands have an inherent mortality rate of ~25%. Additional replicates were used for in vitro experiments when feasible to increase sensitivity of changes to <10 fold signal modulation. |
| Data exclusions | No data were excluded. |
| Replication | Each in vitro experiment was replicated at least twice successfully. Animal experiments were not replicated, but results were concordant. Human studies were not replicated. Efforts to replicate data are described in the manuscript. |
| Randomization | For grouping patients for KM analysis of patients with high hypoxia signature CTCs versus low hypoxia signature CTCs: For each of the patients in the two datasets (GEO GSE144494 and GEO GSE144495) we computed the mean log10(RPM + 1) value of the genes in the hypoxia signature and averaged those means across all the CTCs from that patient. For each dataset we classified those averages as high or low using Otsu's method [Nobuyuki Otsu (1979). "A threshold selection method from gray-level histograms". IEEE Trans. Sys. Man. Cyber. 9 (1): 62–66. doi:10.1109/TSMC.1979.4310076].<br><br>For covariate analysis: To assess breast cancer subtype effect on correlation of CTC hypoxia with overall survival, multivariate Cox proportional hazards modeling was conducted for overall survival after brain metastasis diagnosis by ER, PR, and HER2 status together with Hallmark Hypoxia, Transcription Factor Targets HIF1_Q3, or Transcription Factor Targets HIF1_Q5 geneset expression levels. Results are presented in Supplemental Figure 16.<br><br>No randomization was needed for grouping of mice into experimental groups, as all mice used were female NSG mice, at ~6 weeks of age at experiment initiation. |
| Blinding | Patient blinding was not relevant to the study, as patients were assigned to groups based on level of hypoxic signaling in CTCs.<br>Blinding was not relevant to other experiments in this study, as analysis required knowledge of which group each sample belonged to.<br>However, there was no knowledge of sample characteristics by the research staff who performed RNA-sequencing library preparation. |

# Reporting for specific materials, systems and methods

We require information from authors about some types of materials, experimental systems and methods used in many studies. Here, indicate whether each material, system or method listed is relevant to your study. If you are not sure if a list item applies to your research, read the appropriate section before selecting a response.

## Materials & experimental systems

| n/a | Involved in the study | |
|---|---|---|
| [ ] | [x] | Antibodies |
| [ ] | [x] | Eukaryotic cell lines |
| [x] | [ ] | Palaeontology and archaeology |
| [ ] | [x] | Animals and other organisms |
| [ ] | [x] | Human research participants |
| [ ] | [x] | Clinical data |
| [x] | [ ] | Dual use research of concern |

## Methods

| n/a | Involved in the study | |
|---|---|---|
| [x] | [ ] | ChIP-seq |
| [x] | [ ] | Flow cytometry |
| [x] | [ ] | MRI-based neuroimaging |

## Antibodies

| | |
|---|---|
| Antibodies used | GFP (Abcam ab183734)<br>Ki-67 (Life Technologies 180192Z)<br>Cleaved caspase-3 (Cell Signaling Technology 9664S)<br>HIF1A (Novus NB100-131)<br>HRP anti-rabbit antibody (DAKO)<br>CD45 (R&D Systems, clone 2D1) |

| | |
|---|---|
| | CD66b (AbD Serotec, clone 80H3)<br>EpCAM (Cell Signaling Technology, #5198)<br>Cadherin 11 (R&D Systems, FAB17901G)<br>HER2 (BioLegend, #324410)<br>CD45-TexasRed (BD Biosciences, BDB562279)<br>CD14-TexasRed (BD Biosciences, BDB562334)<br>CD16-TexasRed (BD Biosciences, BDB562320) |
| Validation | Each antibody was validated by the manufacturer for the applications used in this manuscript, per the manufacturer's provided information. |

## Eukaryotic cell lines

Policy information about cell lines

| | |
|---|---|
| Cell line source(s) | HEK293T cells were used and were originally obtained from ATCC. All other cell lines used are circulating tumor cell (CTC) cultures, derived from CTCs isolated from women with metastatic breast cancer using the CTC-iChip as previously described. |
| Authentication | All lines were authenticated by RNA-seq and DNA-seq. |
| Mycoplasma contamination | All lines tested negative for mycoplasma. |
| Commonly misidentified lines (See ICLAC register) | No commonly misidentified cell lines were used in the study. |

## Animals and other organisms

Policy information about studies involving animals; ARRIVE guidelines recommended for reporting animal research

| | |
|---|---|
| Laboratory animals | All mice used in this study were female, NSG mice purchased from Jackson labs, with experiments beginning at 6 weeks of age. They were housed in cages of no more than 4 mice/cage at a temperature of 23+/- 3 degrees C and relative humidity of 30-70%, with 14hr/10hr light/dark cycles. |
| Wild animals | No wild animals were used. |
| Field-collected samples | No field-collected samples were used. |
| Ethics oversight | Institutional Animal Care and Use Committee (IACUC) of the Massachusetts General Hospital (Protocol 2010N000006) |

Note that full information on the approval of the study protocol must also be provided in the manuscript.

## Human research participants

Policy information about studies involving human research participants

| | |
|---|---|
| Population characteristics | All human research participants in this study were women who were at least 18 years old and who carried a diagnosis of breast cancer with brain metastases. |
| Recruitment | Participants were consenting patients being treated at Massachusetts General Hospital and known to treaters to have breast cancer brain metastases. As the patients were not randomized, there is the potential for selection bias, and this was not controlled for in the present study. |
| Ethics oversight | Institutional Review Board approved protocol (DF/HCC 05-300) at Massachusetts General Hospital |

Note that full information on the approval of the study protocol must also be provided in the manuscript.

## Clinical data

Policy information about clinical studies

All manuscripts should comply with the ICMJEguidelines for publication of clinical research and a completedCONSORT checklist must be included with all submissions.

| | |
|---|---|
| Clinical trial registration | *Provide the trial registration number from ClinicalTrials.gov or an equivalent agency.* |
| Study protocol | *Note where the full trial protocol can be accessed OR if not available, explain why.* |
| Data collection | *Describe the settings and locales of data collection, noting the time periods of recruitment and data collection.* |
| Outcomes | *Describe how you pre-defined primary and secondary outcome measures and how you assessed these measures.* |
